# Self-Report of Healthcare Utilization among Community-Dwelling Older Persons: A Prospective Cohort Study

**DOI:** 10.1371/journal.pone.0093372

**Published:** 2014-04-07

**Authors:** Marlies T. van Dalen, Jacqueline J. Suijker, Janet MacNeil-Vroomen, Marjon van Rijn, Eric P. Moll van Charante, Sophia E. de Rooij, Bianca M. Buurman

**Affiliations:** 1 Department of General Practice, Academic Medical Center, University of Amsterdam, Amsterdam, The Netherlands; 2 Department of Internal medicine, Geriatric section, Academic Medical Center, University of Amsterdam, Amsterdam, The Netherlands; Hospital Universitario de Getafe, Spain

## Abstract

**Background:**

Self-reported data are often used for estimates on healthcare utilization in cost-effectiveness studies.

**Objective:**

To analyze older adults’ self-report of healthcare utilization compared to data obtained from the general practitioners’ (GP) electronic medical record (EMR) and to study the differences in healthcare utilization between those who completed the study, those who did not respond, and those lost to follow-up.

**Methods:**

A prospective cohort study was conducted among community-dwelling persons aged 70 years and above, without dementia and not living in a nursing home. Self-reporting questionnaires were compared to healthcare utilization data extracted from the EMR at the GP-office.

**Results:**

Overall, 790 persons completed questionnaires at baseline, median age 75 years (IQR 72–80), 55.8% had no disabilities in (instrumental) activities of daily living. Correlations between self-report data and EMR data on healthcare utilization were *substantial* for ‘hospitalizations’ and ‘GP home visits’ at 12 months intraclass correlation coefficient 0.63 (95% CI; 0.58–0.68). Compared to the EMR, self-reported healthcare utilization was generally slightly over-reported. Non-respondents received more GP home visits (p<0.05). Of the participants who died or were institutionalized 62.2% received 2 or more home visits (p<0.001) and 18.9% had 2 or more hospital admissions (p<0.001) versus respectively 18.6% and 3.9% of the participants who completed the study. Of the participants lost to follow-up for other reasons 33.0% received 2 or more home visits (p<0.01) versus 18.6 of the participants who completed the study.

**Conclusions:**

Self-report of hospitalizations and GP home visits in a broadly ‘healthy’ community-dwelling older population seems adequate and efficient. However, as people become older and more functionally impaired, collecting healthcare utilization data from the EMR should be considered to avoid measurement bias, particularly if the data will be used to support economic evaluation.

## Introduction

Self-reported data on healthcare utilization are often used for estimates of healthcare utilization in cost-effectiveness studies [1]–[3]. Large national studies use surveys to routinely collect these self-reported data [4]–[9]. Self-report is mostly an effective and less time-consuming mode of collecting data on the utilization of healthcare resources compared to collecting data from medical records or administrative claims data. However, while persons at older age and with more disabilities under-report their healthcare utilization [10], [11], research on community-dwelling older persons heavily relies on data solely gathered through self-reported questionnaires [12], which may result in underestimation of health-care cost among older persons.

Various other factors are associated with errors in self-rated outcomes [11], [13]–[15]. For example, inaccuracy increases with longer recall periods [10], [11], [13], [14], [16], [17] and when the frequency of events increases, patients tend to under-report more frequently [10], [14], [16]–[19]. Several studies have compared the accuracy of self-reported with administrative data on healthcare utilization among older persons [11], [13], [22], but they were mostly based on cross-sectional designs.

In longitudinal studies on frail older persons, it is important to note that attrition may be directly related to the primary outcome [20]. Those who do not respond at follow-up are generally older [21]–[25],less educated [8], [25], have lower socioeconomic status [23], live alone [25], have more functional impairments [8], [21], [25], [26], suffer from more comorbidities [21], [22], and are more inaccurate in self-reporting costs compared with participants who completed the study [27]. However, others found no difference between respondents and those who were lost to follow-up, and assumed that attrition was non-selective [9], [28], [29]. However, more research is needed to investigate the relationship between loss to follow-up and healthcare utilization, to study the potential bias in self-reported healthcare utilization data in studies with a longitudinal design.

The objective of this prospective study is therefore to analyze the agreement between older adults’ self-report of healthcare utilization and data obtained from the primary care electronic medical record (EMR) and to study the differences in the healthcare utilization between those lost to follow-up and those who completed the study.

## Methods

### Design and Setting

A prospective cohort study was conducted in seven general practices with a total of 1113 eligible persons aged 70 years and over in and around Amsterdam, the Netherlands. These practices had a mixed population in terms of sex, age, and socio-economic status (SES). The cohort was followed up for 12 months between October 2008 and December 2009.

### Health-care in the Netherlands

In the Dutch health-care system the general practitioner (GP) is the only freely accessible medical professional and people are used to visit their GP if they have a health problem. The GP is the gate-keeper in the healthcare system, controlling access to specialized medical care, and virtually all non-institutionalized citizens are registered with a GP. Therefore the total practice population represents the general population, and information about the wider population is automatically available. For these reasons, in the Netherlands, general practice is the optimal setting for providing information on the populations use of healthcare services [30].

In the Netherlands GPs are financed by a fixed rate based on an average of two office visits per patient per year.

### Ethics Statement

The study was approved by the Medical Ethics Committee of the Academic Medical Center, University of Amsterdam, the Netherlands (protocol ID MEC 10/182).

### Study Population

All community-dwelling persons aged 70 years and over who were registered with one of the participating general practices were selected from the EMR by their GP. Persons were excluded if, according to their GP, they were terminally ill, suffered from dementia, did not understand Dutch, planned to move or spend a long time abroad, or lived in a nursing home. Eligible persons received a letter from their GP with information about the study, along with a written informed consent form, a self-report questionnaire, and a pre-paid envelope. They were invited to fill out the questionnaire themselves, and if they needed help, an informal caregiver was allowed to assist (this assistance was noted on the questionnaire). The recruitment of participants is described in detail elsewhere [31]. All participants were asked to provide written informed consent for data collection and participation in the study on receipt of the study information.

### Measurements

#### Self-reported data

A self-report questionnaire was sent at baseline, and after 3, 6, and 12 months. It comprised demographic data, comorbidities, physical functioning (modified Katz-ADL index score), self-perceived health status, psychological and social functioning (Rand-36), health-related quality of life (EuroQol), and healthcare utilization data [12]. The self-reported data are part of a national database and are publically available [12]. Healthcare utilization data were specified in 1) GP home visit during office hours in the last 3 months and 2) hospital admission in the last 12 months. The number of contacts and the reason for each contact were recorded. Office visits were not gathered since this information would not be of additive value in a cost-effective analysis. Furthermore, the original study objective was to evaluate acute/un-planned healthcare utilization of community dwelling older persons, which did not include office visits.

#### Electronic medical records

EMR data were used to calculate healthcare utilization. Healthcare utilization data from October 2008 to December 2009 were extracted from the EMR at the GP’s office by two independent researchers (MvD and JS). First, billing data were searched for home visits. Hospital admissions were then extracted from the patient’s history, and the correspondence section in the EMR was searched for discharge letters. All hospital admissions were recorded. No difference was made between an admission for one day without an overnight stay and an admission of one or more overnight stays. The number of hospitalization days was not recorded. If a GP contact was followed by a hospital admission on the same day, only the hospital admission was recorded, because it was difficult to discriminate between the number of home visits on the same day before hospitalization or between in or out-of-hours GP-home visits.

#### Linking self-reported data to the EMR

The self-reported data on healthcare utilization were linked to the data derived from the EMR using the date on which the questionnaire was filled out. To analyze the agreement between self-report data and EMR data for GP home visits, questionnaires returned after 3, 6, and 12 months were used. To analyze the agreement between self-report data and EMR data for hospital admission, questionnaires returned after 12 months were used.

### Non-respondents at Baseline

Previous studies suggested that non-respondents are more often cognitively and functionally impaired and have a higher mortality rate than responding participants, and therefore are often considered a high-risk population [7], [32], [33]. Therefore, non-respondents may use healthcare services more frequently.

Non-response is a collective term for persons (called non-respondents or non-participants) who are invited to participate in a study but do not do so [34]. To explore whether non-respondents were more often functionally and/or cognitively impaired and used healthcare services more frequently, a sample of randomly selected non-respondents and a sample of randomly selected participants were invited to participate in a single home visit conducted by a trained research nurse, on behalf of their own GP. Informed consent was obtained before the interview took place. During this home visit, the same baseline self-report questionnaire was conducted along with a Mini-Mental State Examination (MMSE) [35] in both groups. The healthcare utilization computed from the EMR over the year 2009 was compared between both groups.

### Respondents Lost to Follow-up

Self-reported baseline characteristics and healthcare utilization computed from the EMR over the year 2009 were compared between participants who completed follow-up and those who were lost to follow-up after 12 months. Participants who died or were institutionalized were analyzed separately, with the assumption that this group was prone to higher levels of healthcare utilization in the period before death or institutionalization [36]. Older persons lost to follow-up for other reasons were defined as those declining further participation, not able to be contacted at follow-up or lost to follow-up for other reasons.

### Statistical Methods

Baseline descriptive statistics were used. Differences in baseline characteristics and healthcare utilization between participants and non-respondents, and between participants who completed follow-up and those lost to follow-up were estimated using a Mann-Whitney U test for continuous, nonparametric data or a Chi-square test for ordinal variables. Overall, a p-value of <0.05 was considered statistically significant.

Data on healthcare utilization were assessed as the number of contacts with healthcare services. The number of self-reported home visits and hospital admissions were compared with the number of home visits and hospital admissions derived from the EMR. To evaluate the agreement between self-report of healthcare utilization and data from the EMR, 3 groups were identified: (1) Under-reporting (patient reported less utilization in comparison to data retrieved from the EMR), (2) Exact agreement (self-reported and the EMR agreed on the volume of utilization), (3) Over-reporting (participant reported more utilization than could be retrieved from the EMR).

Levels of agreement between self-reports and the EMRs were assessed for each follow-up time using absolute agreement and intraclass correlation coefficients (ICCs)[37]. The benchmark for determining the closeness of the comparison of the ICC was based on Landis and Koch’s scale [38]. The strength of agreement was <0.00 (poor), 0.00–0.20 (slight), 0.21–0.40 (fair), 0.41–0.60 (moderate), 0.61–0.80 (substantial) and 0.81–1.00 (almost perfect) [38].

Finally, healthcare utilization and participation time in the study were plotted against age and number of disabilities in (instrumental) activities of daily living ((I)ADL). Analyses were performed with the Statistical Package for the Social Sciences (SPSS) version 18.0.

## Results

### Eligible Persons and Baseline Characteristics

**Figure 1:**
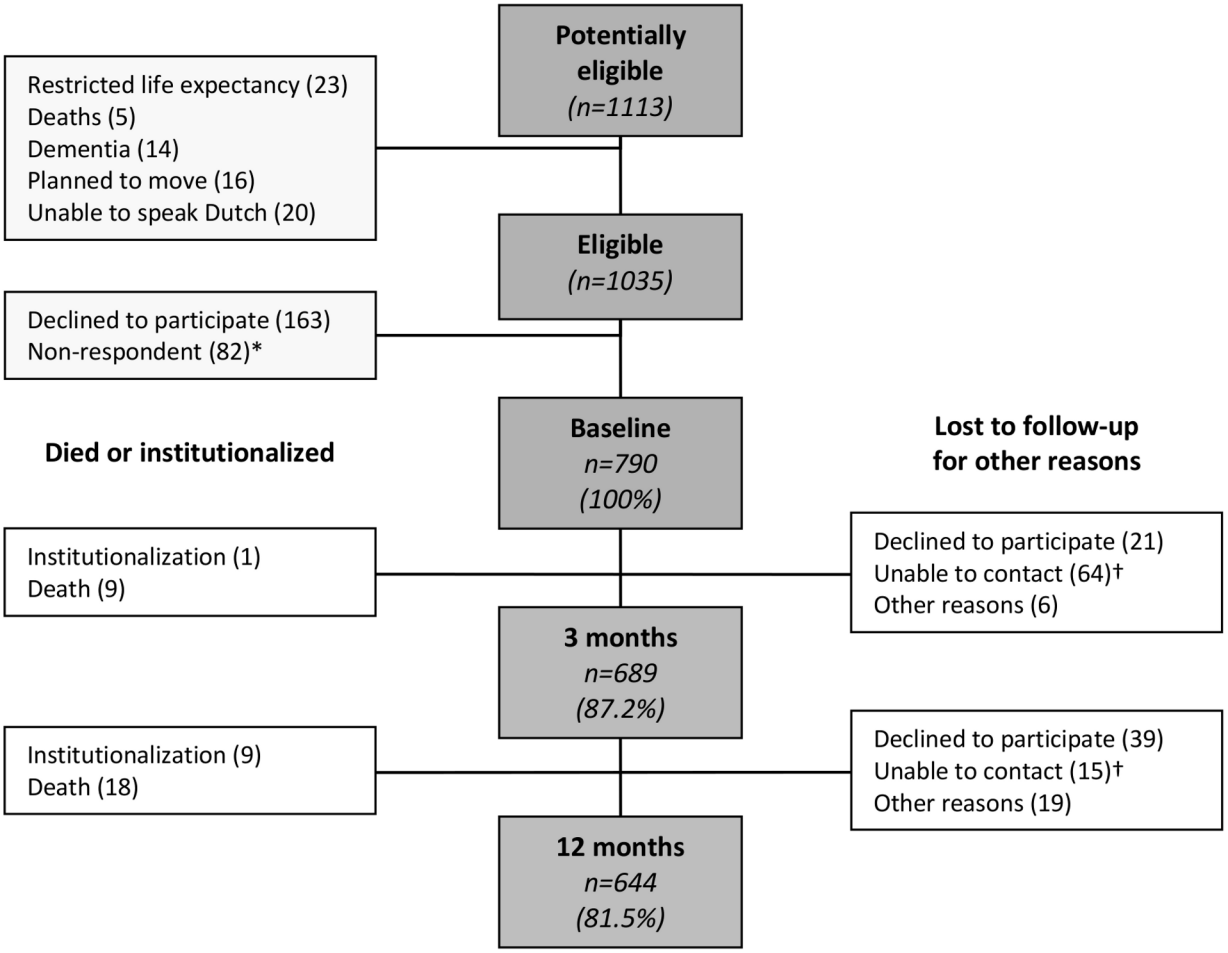
Flowchart. *Non-respondents were those we were unable to contact at baseline. Those who are denoted as ‘unable to contact’ are those persons not responding at follow-up. † These numbers represent a variable group of persons since persons who did not respond at three months follow up, might respond at a later follow-up moment.

**Figure 2:**
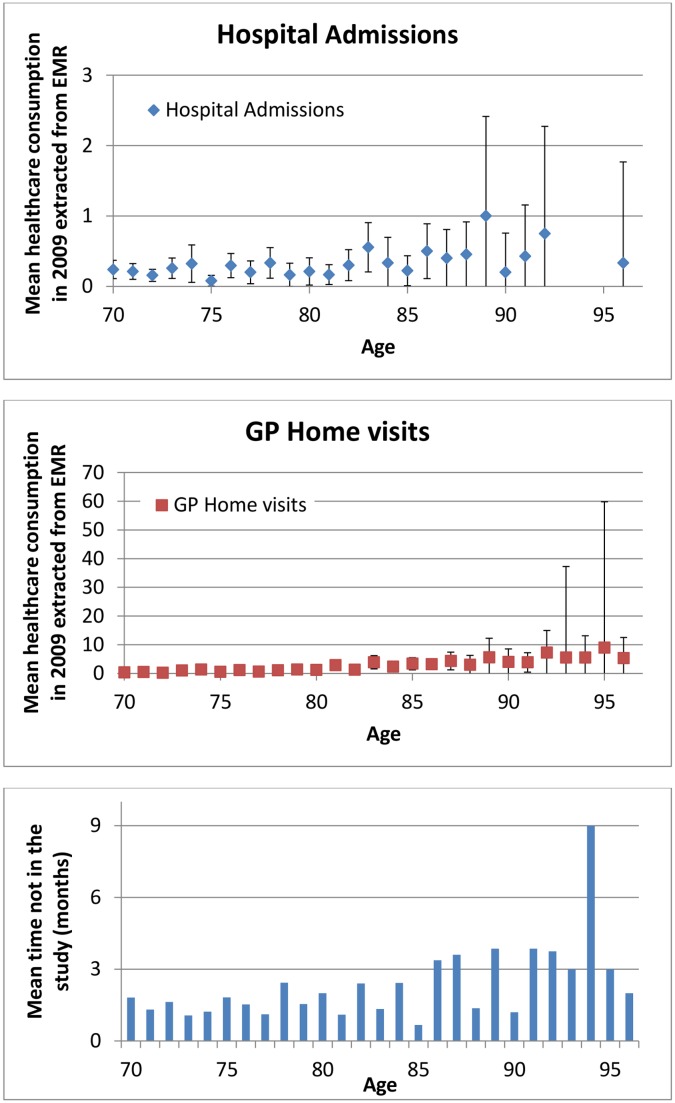
Mean healthcare utilizaton in 2009 extracted from the EMR and the mean time not in the study with increasing age (years) (n = 790). Mean healthcare consumption with 95%CI.

**Figure 3:**
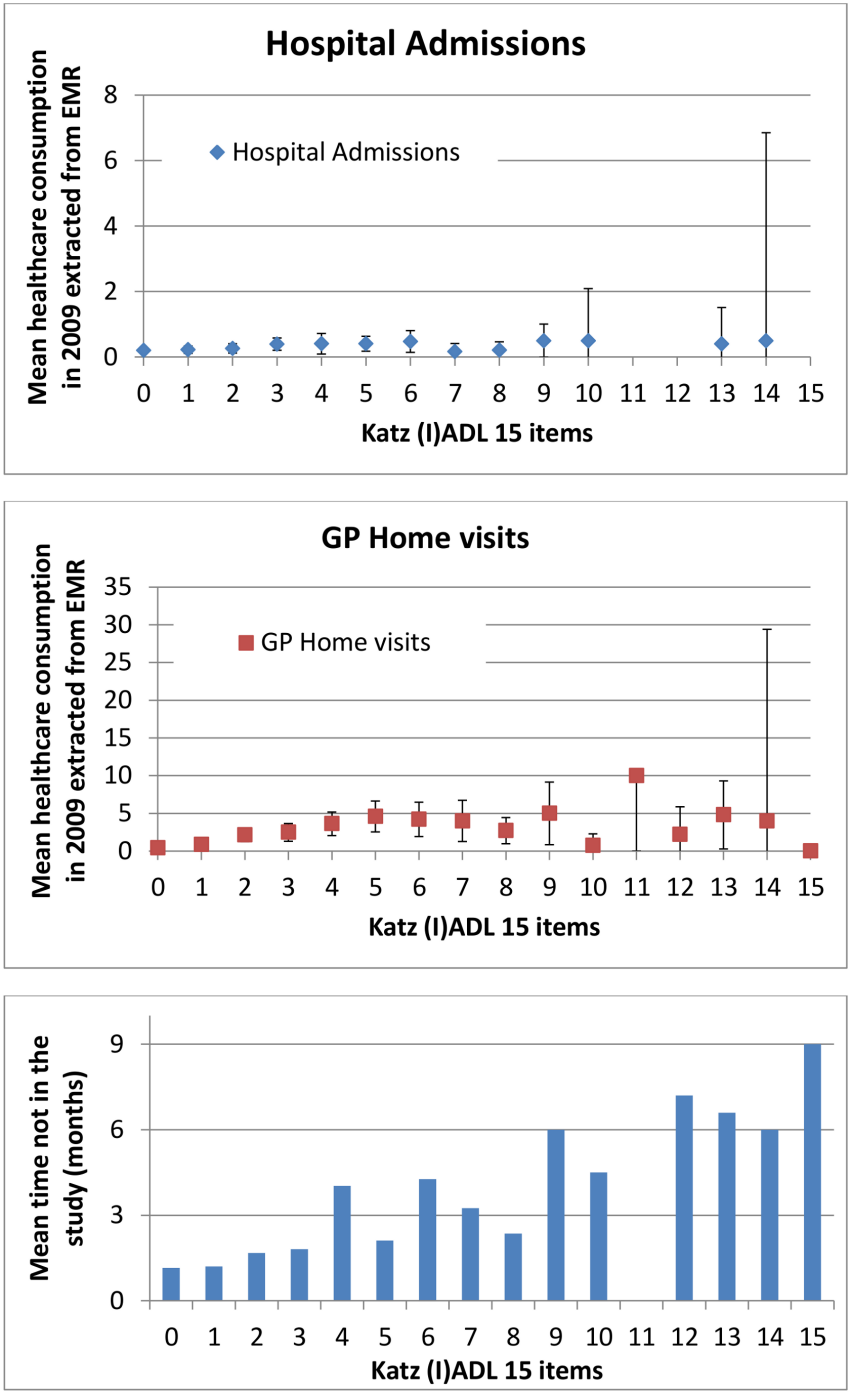
Mean healthcare utilizaton in 2009 extracted from the EMR and the mean time not in the study with increasing number of (I)ADL disabilities (n = 790). Mean healthcare consumption with 95%CI.

The cohort comprised 1113 persons. Reasons for exclusion and non-response are shown in figure 1. In total, 790 (76.3%) participants returned the baseline questionnaire and were included. At baseline, the median age was 75 years (IQR 72–80), 56.7% were female, 55.8% participants had no (I)ADL disabilities and 16.2% reported memory problems (Table 1). A total of 644 (81.5%) participants returned the questionnaire after 12 months. The number of persons lost to follow-up due to death or institutionalization ranged from 9.1% at 6 months to 11.0% at 12 months, calculated over the total number of persons lost to follow-up. Figure 2 shows the mean healthcare utilizaton in 2009 extracted from the EMR and the mean time not in the study with increasing age. Figure 3 demonstrates the mean healthcare utilizaton in 2009 extracted from the EMR and the mean time not in the study with increasing number of (I)ADL disabilities.

**Table 1:** Description of baseline characteristics.

	Participants	Missing*
	(n = 790)*	%
Age, y, median (IQR)	75 (72–80)	0.0
Female	56.7	0.0
Born in the Netherlands	85.4	0.1
Level of education		1.0
Primary school or less	22.4	
Secondary education or vocational school	61.9	
College or university	14.8	
Socioeconomic Status		0.0
Low (≤1SD)	32.7	
Intermediate	42.8	
High (≥1SD)	24.4	
Marital status		0.4
Married/Living together consistently or with child	51.8	
Divorced/Widow/Not married	48.2	
Living situation		1.6
Independent	92.7	
Home for elderly	6.1	
Living with friend or family	1.2	
Modified Katz ADL (15 items)		2.9
0	55.8	
1–2	20.6	
≥3	23.6	
Depressive symptoms (GDS-2)	15.6	5.7
Comorbidities:		0.0
0	14.3	
1	22.2	
≥2	63.5	
Self-reported memory problems	16.2	2.3
Polypharmacy (≥3)	60.3	6.2
Self-reported health status compared to 1 year ago		1.5
Better/Same/Worse	9.8/60.4/29.8	
Limitations of social activities		4.6
Constant/Sometimes/Never	13.8/18.8/67.4	

*Values are percentages unless otherwise noted.

*IQR  =  interquartile range; SD  =  standard deviation; ADL  =  Activities of Daily Living; GDS  =  Geriatric Depression Scale (GDS-2).*

### Agreement between Self-reported Healthcare Utilization and EMRs

**Figure 4:**
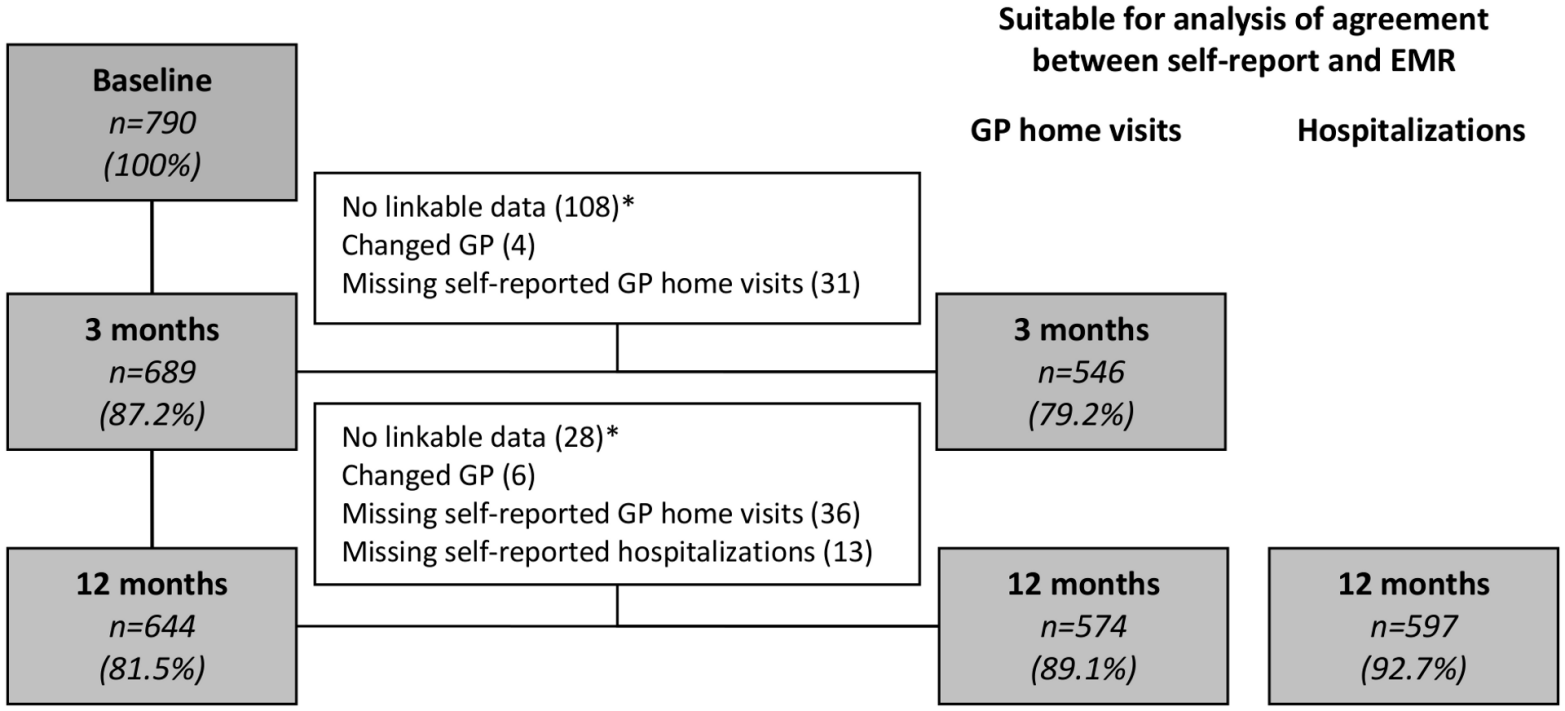
Flowchart data retrieval. *The period of data retrieval did not cover the recall period.

Figure 4 shows all persons included in the analysis based on possible data linkage. The agreement between both data sources was 86.1% for home visits, and 86.9% for hospital admission. The strength of agreement (ICC) between both data sources at 12 months was 0.63 (95% CI; 0.58–0.68) for both home visits and for hospitalization, *substantial* in both cases. Agreement between both data sources after three and six months was similar (data not shown). In general, participants slightly over-reported their healthcare utilization. Table 2 and 3 show the healthcare utilization (GP home visits and hospital admission) according self-reported data and EMR at twelve months. Table 4 shows the prevalence and measures of agreement of healthcare utilization according self-reported data and EMR at twelve months. Characteristics of persons under- or over-reporting versus agreement between self-report and EMR, are displayed in Table S1.

**Table 2:** The numbers of self-reported GP-homevisits and GP-homevisits according to the Electronic Medical Record at 12 months.

Self-reported GP home visits	Number of GP home visits according to EMR (n = 574)					
	0	1	2	3	≥4	Total
0	461	13	3	1	0	478
1	22	21	4	2	2	51
2	7	6	7	1	0	21
3	3	0	5	2	1	11
≥4	2	3	1	4	3	13
Total	495	43	20	10	6	574

**Table 3:** The numbers of self-reported hospitalization and hospitalization according to the Electronic Medical Record at 12 months.

Self-reported hospitalizations	Number of hospitalizations according to EMR (n = 597)					
	0	1	2	3	≥4	Total
0	465	21	5	0	0	491
1	23	46	5	0	0	74
2	8	10	7	0	0	25
3	1	1	1	0	0	3
≥4	1	1	0	1	1	4
Total	498	79	18	1	1	597

**Table 4:** Agreement between self-reported and Electronic Medical Record healthcare utilization at 12 months.

	12 months
GP home visit	(n = 574)†
≥1 visit according to EMR	79 (13.8)
Under-reporting	27 (4.7)
Agreement	494 (86.1)
Those with both responses zero, %*	461 (93.3)
Over-reporting	53 (9.2)
Absolute difference, median (IQR)	0 (0)
ICC (95% CI)	0.63 (0.58–0.68)
**Hospitalization**	**(n = 597)**
≥1 hospital admission according to EMR	99 (16.6)
Under-reporting	31 (5.2)
Agreement	519 (86.9)
Those with both responses zero, %*	465 (89.6)
Over-reporting	47 (7.9)
Absolute difference, median (IQR)	0 (0)
ICC (95% CI)	0.63 (0.58–0.68)

† *Values are numbers and percentages between brackets, unless otherwise noted.*

*self-reported data and EMR data indicated no event.

*GP  =  general practitioner; EMR  =  electronic medical record; IQR  =  inter quartile range; ICC  =  intraclass correlation coefficients; CI  =  confidence interval.*

### Non-respondents

For the non-respondent analysis, 103 home visits were conducted. Of those persons, 32 were non-respondents and 71 responded to our surveys. Non-respondents had a lower socioeconomic status (SES) (p<0.05), a lower scores on the MMSE (p<0.01) and received more GP home visits compared to respondents (p<.05). There were no differences in hospitalization between both groups (p = 0.788) (Table S2).

### Healthcare Utilization of Participants Compared to those Lost to Follow-up

At 12 months, those who died or were institutionalized were older (p<0.001), more disabled in (I)ADL (p<0.001) and had more other risk factors for functional decline at baseline. Those lost to follow-up for other reasons had more often a non-Caucasian ethnic background (p<0.001), a lower socioeconomic status (p = 0.002), were more disabled in (I)ADL (p<0.001) and were less able to participate (p<0.001) (Table 5).

**Table 5:** Baseline characteristics of participants compared to those lost to follow-up (percentages).

	Participants	Died or institutionalized	Lost to follow-up for other reasons
Variable	(n = 644)†	(n = 37)†	(n = 109)†
Age, y, median (IQR)	75 (72–80)	**82 (77–88)** ***	75 (72–80)
Female	55.7	67.6	58.7
Born in the Netherlands	88.3	81.1	**69.7** ***
Socioeconomic status *low* (≤1SD)	29.5	**48.6** *	**46.2** **
Living situation *independent*	93.5	**77.1** ***	**92.6** *
Modified Katz (I)ADL (15 items)			
0	60.1	**15.2** ***	**43.5** ***
1–2	21.1	**27.3**	**15.7**
≥3	18.8	**57.6**	**40.7**
Multimorbidity, ≥2 comorbidities	62.6	78.4	64.2
Depressive symptoms (GDS-2)**	13.3	**40.6** ***	**21.4** *
Self-reported memory problems	14.8	**32.4** **	19.0
Polypharmacy (≥3)	59.7	76.5	58.3
Self-reported health status *worse* compared to 1 year ago	26.7	**56.8** ***	**39.3** *
Hindrance of social activities *constant*	10.9	**42.4** ***	**22.7** ***

† *Values are percentages unless otherwise noted.*

*The Mann-Whitney U test was used for continuous variables. The chi-square test was used for binary or ordinal variables.*

*p≤0.05.

**p≤0.01.

***p≤0.001 compared to respondents. Significant differences are marked in bold.

Of all 790 persons included in this study, 19.5% were hospitalized and 32.7% received home visits in the year 2009 according to the EMR. Table 6 displays the healthcare utilization of respondents, those who died or were institutionalized, and those lost to follow-up for other reasons. Persons who died or were institutionalized had significantly higher overall levels of healthcare utilization compared to respondents and those lost to follow-up for other reasons (p<0.001) for both GP home visits and hospitalizations. This finding was already apparent after 3 months’ follow-up (data not shown). Those lost to follow-up for other reasons had more home visits by their GP after 12 months (p = 0.003). This finding was consistent, though not significant, with those at 3 and 6 months follow-up.

**Table 6:** Healthcare utilization of participants compared to those lost to follow-up.

	Participants	Died or institutionalized	Lost to follow-up for other reasons
Healthcare utilization in 2009	(n = 644)†	(n = 37)†	(n = 109)†
GP home visits			
0	71.0	**27.0** ***	**59.6** **
1	10.4	**10.8**	**7.3**
≥2	18.6	**62.2**	**33.0**
Hospital admissions			
0	83.1	**35.1** ***	80.7
1	13.0	**45.9**	13.8
≥2	3.9	**18.9**	5.5

† Values are percentages.

The chi-square test was used for ordinal variables.

*p≤0.05.

**p≤0.01.

***p≤0.001 compared to respondents. Significant differences are marked in bold.

## Discussion

### Main Findings

In this prospective cohort study among 790 community-dwelling older persons, correlations between self-report data and EMR data on healthcare utilization were *substantial* for ‘hospitalizations’ and ‘GP home visits’ at 12 months. Healthcare utilization was slightly over-reported compared to data based on the EMR. Non-respondents had a lower socioeconomic status, a lower MMSE score at baseline, and received more GP home visits. Participants who died or were institutionalized were older and more often functionally impaired, and utilized more healthcare services. Participants lost to follow-up for other reasons only received more home visits by GPs. This study showed that with increasing age and disabilities in daily functioning, healthcare utilization increased and participation time in the study decreased.

### Comparison with the Literature

Our findings of over-reporting of healthcare utilization compared to the EMRs contradict some studies [16], [17], but are consistent with others [11], [15]. Raina et al. found that patients aged 65 years and older (n = 1,038) over-reported hospitalizations over 12 months in 3.3% of the cases and under-reported hospitalizations in 3.1% [15]. In our study these rates were 8.0% and 5.2%, respectively. Raina et al. observed a patient agreement about hospitalization in 13.5% at baseline interviews, compared to 10.4% after 12 months in our study. Wolinsky et al. found comparable measures for misreporting hospitalizations [11].

We did not find any literature on healthcare utilization among non-respondents.

In our study, 18.5% of persons were lost to follow-up. Participants who had died or were institutionalized were older, more often disabled, more often depressed, reported more often memory problems, had worsened health status compared to a year ago and were less able to participate at baseline. Participants lost to follow-up for other reasons more often had a non-Caucasian ethnic background, had a lower socioeconomic status, were more often disabled and were less able to participate. These findings are in accordance with the literature [8], [21]–[26], although some authors found no difference between responders and those lost to follow-up [28] or assumed nonselective loss to follow-up [9], [29].

Older persons who died or were institutionalized appeared to have significantly higher levels of healthcare utilization. Those lost to follow-up for other reasons received more home visits, though no differences were found in hospitalizations. Hoogendoorn et al. compared self-report versus care provider registration of healthcare utilizations and its impact on cost and cost-utility. They found that the degree of under-reporting was independently associated with loss to follow-up and total costs, showing that those lost to follow-up had larger differences in costs between registrations and self-reports compared with full participants [27].

### Strengths and Limitations

This study has several limitations. Firstly, using the EMR as the reference standard is not without its own drawbacks [10], [16], [18], [39]. For example, a substantial proportion (9.5%) of patients consume healthcare outside the urban healthcare system, suggesting that this use may not be captured in the EMR [10]. GPs also tend to under-report repeated consultations for the same problem [40]. However, Dendukuri et al. showed that claims databases had the greatest validity [41]. Some persons were repeatedly hospitalized, though the GP was sometimes notified only once. Furthermore, GP contacts on the same day prior to hospitalization were often omitted when extracting healthcare utilization from the EMR, resulting in a potential underestimation of GP home visits. Moreover, when a patient is dismissed from a hospital, it can take some time before the hospitalization can be found in the EMR.

Secondly, as a measure of healthcare utilization, only hospitalizations and GP home visits were taken into account. When this study was first conducted, information on emergency department (ED) visits and contact with GPs during out-of-hours were also collected from participants. However, the GP was often not separately informed about ED visits, especially if a patient was hospitalized afterwards, and therefore this contact was not retrieved from the EMR. However, it is important to note that 70% of ED visits by older people is followed by a hospital admission [42]. Furthermore, it turned out that the prevalence of out-of-hours GP contacts in our population was very low; therefore, these contacts were left out of the analyses too.

Thirdly, participants may have found it difficult to discriminate between home visits paid by GPs and registered nurses, or between visits during in or out-of-hours, yielding lower agreement measures. Furthermore, GP home visits were not counted in the EMR if they were on the same day as a hospital admission, leading to an underestimation of the costs made by GPs and to an apparent overestimation of self-reported home-visits.

Most studies on the agreement of self-reports have cross-sectionally examined populations. The strength of our study lies in the prospective design and our additional non-respondent analysis, comparing healthcare utilization patterns of respondents with non-respondents and those who died, institutionalized or otherwise lost to follow-up.

### Implications

In our study, correlations between self-report and EMR data for GP home visits and hospitalizations were nearly identical. The impact of that difference on, for example, cost-effectiveness studies should be taken into consideration. For example, Hoogendoorn et al. found that despite the almost perfect agreement on hospitalizations and hospital days, the cost difference was highest for this type of care, approximately 390 euros [27].

This research also shows that the inclusion of data from older persons lost to follow-up during prospective cohort studies is important. Those who die or are institutionalized tend to use more healthcare services than those remaining in the study. Those lost to follow-up for other reasons also tend to place a larger burden on more intensive primary healthcare services than those remaining in the study. Therefore, the more substantial the loss to follow-up becomes, the more need there may be to consider data-collection on health services utilization from the EMR.

## Conclusion

Self-report of hospitalizations and GP home visits in a broadly ‘healthy’ community-dwelling older population seems adequate and efficient. However, as people become older and more functionally impaired, collecting healthcare utilization data from the EMR should be considered to avoid measurement bias, particularly if the data will be used to support economic evaluation.

### What is New?

- Self-report of hospitalizations and GP home visits in a broadly ‘healthy’ community-dwelling older population seems adequate and efficient.
- However, as people become older and more functionally impaired, collecting healthcare utilization data from the electronic medical records should be considered to avoid measurement bias, particularly if the data will be used to support economic evaluation.
- Older persons who died or were institutionalized during follow-up tended to use more healthcare services than participants.
- Those who were lost to follow-up for other reasons tended to receive more GP home visits than those remaining in the study.

## Supporting Information

Table S1 Patient characteristics versus reporting agreement.
(DOC)

Table S2 Non-respondent analysis.
(DOC)
