# Supplementary material for: Self-Report of Healthcare Utilization among Community-Dwelling Older Persons: A Prospective Cohort Study
Source: PLoS One. 2014 Apr 7;9(4):e93372. doi: 10.1371/journal.pone.0093372 (PMC3977826; doi:10.1371/journal.pone.0093372)
Supplement: Table S1 — Patient characteristics versus reporting agreement. (DOC) [file pone.0093372.s001.doc]

**Supporting Information S1**

**Table S1 Patient characteristics versus reporting agreement**

|  | GP Home visits  (n=546) | | | Hospital admissions  (n=597) | | |
| --- | --- | --- | --- | --- | --- | --- |
|  | 3-month recall ‡ | | | 12-month recall‡ | | |
| Variable | Under-reporting (n=37)† | Agreement (n=442)† | Over-reporting (n=67)† | Under-reporting (n=31)† | Agreement (n=518)† | Over-reporting (n=48)† |
| Age, y, median (IQR) | **81 (74-85)** | **74 (72-79)** | **78 (74-83)***** | 77 (72-83) | 75 (71-78) | 73 (71-79) |
| Female | 70.3 | 54.5 | 70.1 | 58.1 | 56.2 | 45.8 |
| Born in the Netherlands | 86.5 | 88.7 | 76.1 | 80.6 | 89.4 | 1.7 |
| Socioeconomic status *low* (≤1SD) | **48.6** | **27.9** | **41.8***** | 35.5 | 28.0 | 20.8 |
| Living situation *independent* | 80.0 | 93.2 | 85.1 | 93.5 | 94.0 | 93.8 |
| Questionnaire filled out by proxy | 63.9 | 84.0 | 64.6 | 16.1 | 19.6 | 15.2 |
| Modified Katz ADL index (15 items) |  |  |  |  |  |  |
| 0 | **28.6** | **60.4** | **34.8***** | 54.8 | 58.9 | 44.7 |
| 1-2 | **14.3** | **21.4** | **28.8** | 19.4 | 21.8 | 29.8 |
| ≥3 | **57.1** | **18.2** | **36.4** | 25.8 | 19.3 | 25.5 |
| Frequency of visits, median(IQR) | **1 (1-3)** | **0 (0-0)** | **0 (0-1)***** | **1 (1-2)** | **1 (1-1)** | **0 (0-1)***** |
| Multimorbidity, ≥2 comorbidities | **73.0** | **55.1** | **77.6***** | 71.0 | 64.4 | 75.0 |
| Depressive symptoms (GDS-2) | **30.6** | **12.3** | **32.3***** | 0 | 10.3 | 18.8 |
| Self-reported memory problems | **32.4** | **13.8** | **24.2**** | 16.1 | 18.4 | 10.4 |
| Polypharmacy (≥3) | **82.9** | **56.5** | **70.8**** | **77.4** | **60.9** | **80.4**** |
| Self-reported health status *worse* compared to 1 year ago | **43.2** | **20.8** | **50.0***** | **29.0** | **29.1** | **41.7***** |
| Hindrance of social activities *constant* | **43.2** | **10.1** | **24.2***** | 9.7 | 11.1 | 20.8 |

*†Values are percentages unless otherwise noted*

*‡Self-report at three months follow-up was used for GP Home visits and twelve months follow-up for hospital admissions.*

*The Kruskal-Wallis test was used for continuous variables. The chi-square test was used for binary or ordinal variables.
*p<0.05, **p<0.01, ***p<0.001; Significant differences are marked in bold.
IQR = interquartile range; SD = standard deviation; ADL = Activities of Daily Living;*

*GDS = Geriatric Depression Scale .*
