# Supplementary material for: Self-Report of Healthcare Utilization among Community-Dwelling Older Persons: A Prospective Cohort Study
Source: PLoS One. 2014 Apr 7;9(4):e93372. doi: 10.1371/journal.pone.0093372 (PMC3977826; doi:10.1371/journal.pone.0093372)
Supplement: Table S2 — Non-respondent analysis. (DOC) [file pone.0093372.s002.doc]

**Supporting Information S2**

**Table S2** Non-respondent analysis

|  | Respondents (n=71) † | Non-respondents (n=32) † |
| --- | --- | --- |
| Age, y, median (IQR) | 77 (73-82) | 76 (72-83) |
| Female | 66.2 | 50.0 |
| Born in the Netherlands | 81.7 | 75.0 |
| Socioeconomic status  low (≤1SD)  intermediate  high (≥1SD) | 38.6  11.4  50.9 | **59.4*****  **18.8**  **21.9** |
| Modified Katz ADL index (15 items) |  |  |
| 0 | 32.4 | 18.8 |
| 1-2 | 32.4 | 34.4 |
| ≥3 | 35.2 | 46.9 |
| Depressive symptoms (GDS-2) | 11.4 | **48.4******* |
| Self-reported memory problems | 36.6 | 48.4 |
| Polypharmacy (≥3) | 76.1 | 71.0 |
| MMSE Score, Median (IQR) | 27 (25-29) | **25 (19-28)****** |
| GP home visits, Median (IQR) | 0 (0-2) | **1 (0-6)***** |
| Hospitalizations, Median (IQR) | 0 (0-0) | 0 (0-0) |

*†Values are percentages unless otherwise noted*

*The Mann-Whitney U test was used for continuous variables. The chi-square test was used for binary or ordinal variables and the Fisher’s Exact test was used when values were <10.
*p<0.05, **p<0.01, ***p<0.001
IQR = interquartile range; SD = standard deviation; ADL = Activities of Daily Living; GDS = Geriatric Depression Scale.*
